# Rapid response to high-dose Anakinra in refractory SLE-associated Haemophagocytic Lymphohistiocytosis: a case report

**DOI:** 10.1093/omcr/omag130

**Published:** 2026-09-25

**Authors:** S Jain, N Soe, K Sandhu, J McNally

**Affiliations:** Department of Rheumatology, Royal Berkshire Hospital, London Road, Reading, Berkshire, RG1 5AN, United Kingdom; Department of Rheumatology, University Hospital Coventry and Warwickshire, Clifford Bridge Road, Coventry, Warwickshire, CV2 2DX, United Kingdom; Department of Rheumatology, Royal Berkshire Hospital, London Road, Reading, Berkshire, RG1 5AN, United Kingdom; Department of Rheumatology, Royal Berkshire Hospital, London Road, Reading, Berkshire, RG1 5AN, United Kingdom

**Keywords:** rheumatology, haematology

## Abstract

Haemophagocytic Lymphohistiocytosis (HLH) causes life-threatening hyperinflammation and has conventionally been treated with glucocorticoids plus additional immunosuppression such as ciclosporin and anakinra. We describe the case of a 47-year-old female who presented to a DGH with HLH secondary to both Systemic Lupus Erythematosus (SLE) and Covid infection. Her disease relapsed despite an initial response to first-line treatment including glucocorticoids, ciclosporin, intravenous immunoglobulins (IVIG) and conventional doses of anakinra. She subsequently responded to a higher dose of anakinra, resulting in sustained remission. This case illustrates the potentially life-saving difference between conventional and higher anakinra doses in severe HLH- an observation that is consistent with other reported cases of HLH with different triggers. We argue, therefore, for high-dose anakinra to be used first-line in refractory HLH, given the high mortality of the disease, while also carefully monitoring for infections post-interleukin-1-blockade. This adds weight to the shifting national treatment paradigm in this direction.

## Introduction

Haemophagocytic Lymphohistiocytosis (HLH) is a life-threatening hyperinflammatory syndrome driven by uncontrolled proliferation of T-lymphocytes and macrophages, leading to cytokine storm and multi-organ failure (EULAR/ACR 2022, GIRFT-HLH 2025 guidelines). Patients present with fever, pancytopenia and neurological disturbance, with one-year mortality of 50% even with treatment [1, 2]. Triggers include infection, malignancy, autoimmune and haematological disease. Glucocorticoids are used as first-line treatment, combined with second-line options such as anakinra, ciclosporin, etoposide and intravenous immunoglobulin (IVIG) [3]- as outlined in cross-speciality national guidance [1, 3].

Systemic lupus erythematosus (SLE) is a multisystem autoimmune disease with estimated global prevalence of 44/100000, which can predispose to life-threatening conditions including cervical artery dissection [4], catastrophic antiphospholipid syndrome [5] and indeed HLH. In adults with SLE, the incidence of HLH ranges from 0.9% to 4.6% [6].

This case report explores the use of high-dose anakinra in refractory SLE-induced HLH, with Covid as an additional trigger, highlighting its potential as first-line therapy. We review the argument for more rapid dose escalation in such cases.

## Case report

A 47-year-old female with SLE maintained on Mycophenolate 1 g twice-daily and Prednisolone 2.5 mg daily presented to a district general hospital (DGH) with cough, fever and unsteady gait. There was no hepatosplenomegaly, lymphadenopathy or rash. She had borderline pancytopenia, C-reactive protein (CRP) 35 mg/l, ferritin 4649ug/l, low C3 and normal C4. Serology revealed speckled ANA 1:640, dsDNA 21, positive anti-U1RNP and anti-Ro. She tested positive for Covid, was treated with Remdesivir and discharged.

She was readmitted on day 2 with persistent fever (39.2°C) and hypotension (80/40 mmHg) and managed empirically on intensive care with intravenous fluids and broad spectrum antibiotics. Investigations showed Ferritin 12 737 rising to 17301ug/L, triglycerides 4.8 mmol/l, lactate dehydrogenase 573 U/l, CRP 65 mg/l, erythrocyte sedimentation rate 8 mm/hr, alanine aminotransferase 74 U/l, haemoglobin 79 g/l, neutrophils 1.6 × 10^*^9/l, lymphocytes 0.3 × 10^*^9/l, fibrinogen 1.6 g/l and normal renal function. CT head and lumbar puncture were unremarkable, ruling out central nervous system infection. CT chest/abdomen/pelvis excluded solid organ malignancy. Her remaining viral screen was negative.

**Figure 1:**
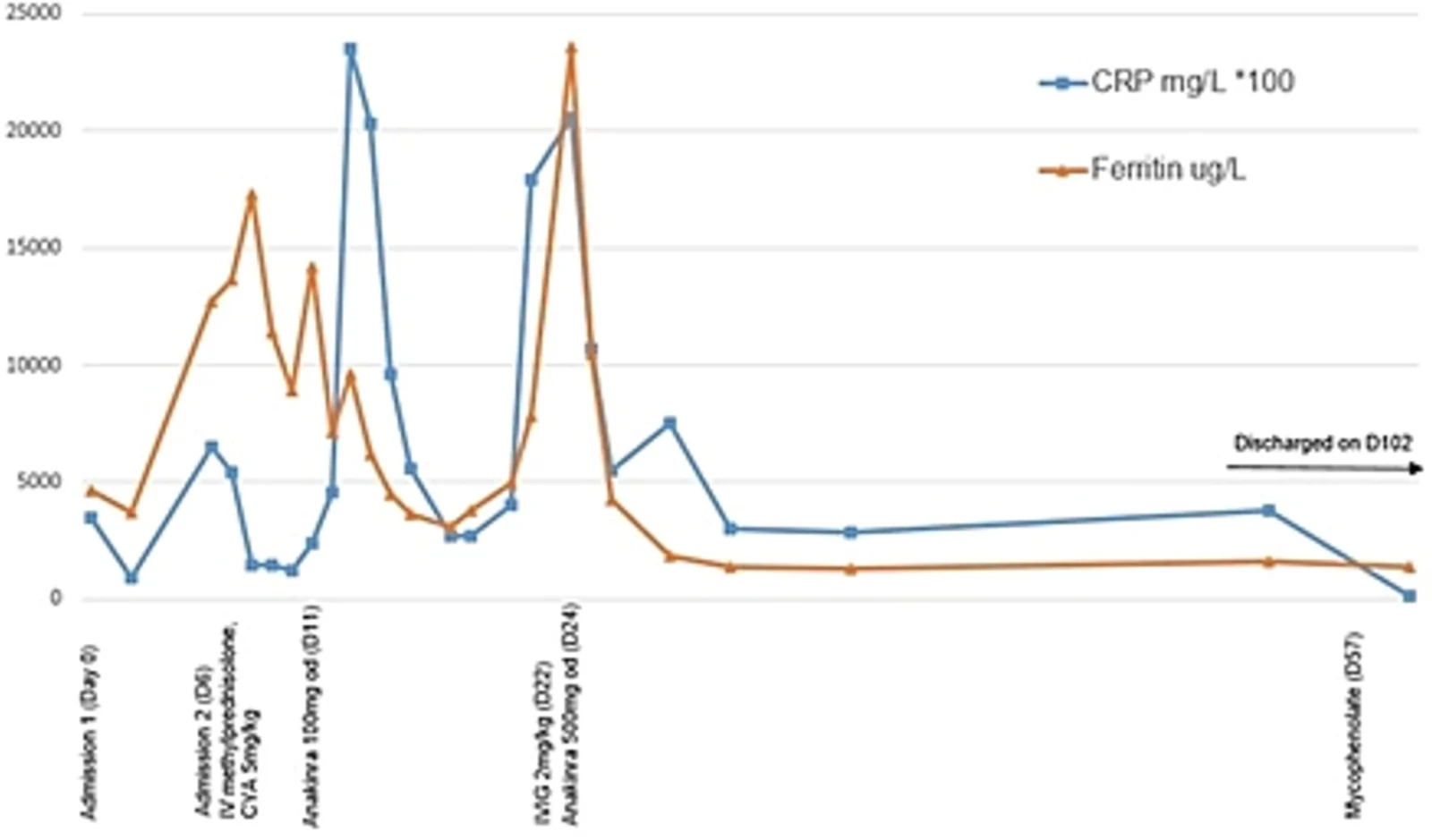
Response of HLH biomarkers to different treatments over the course of admission.

**Figure 2:**
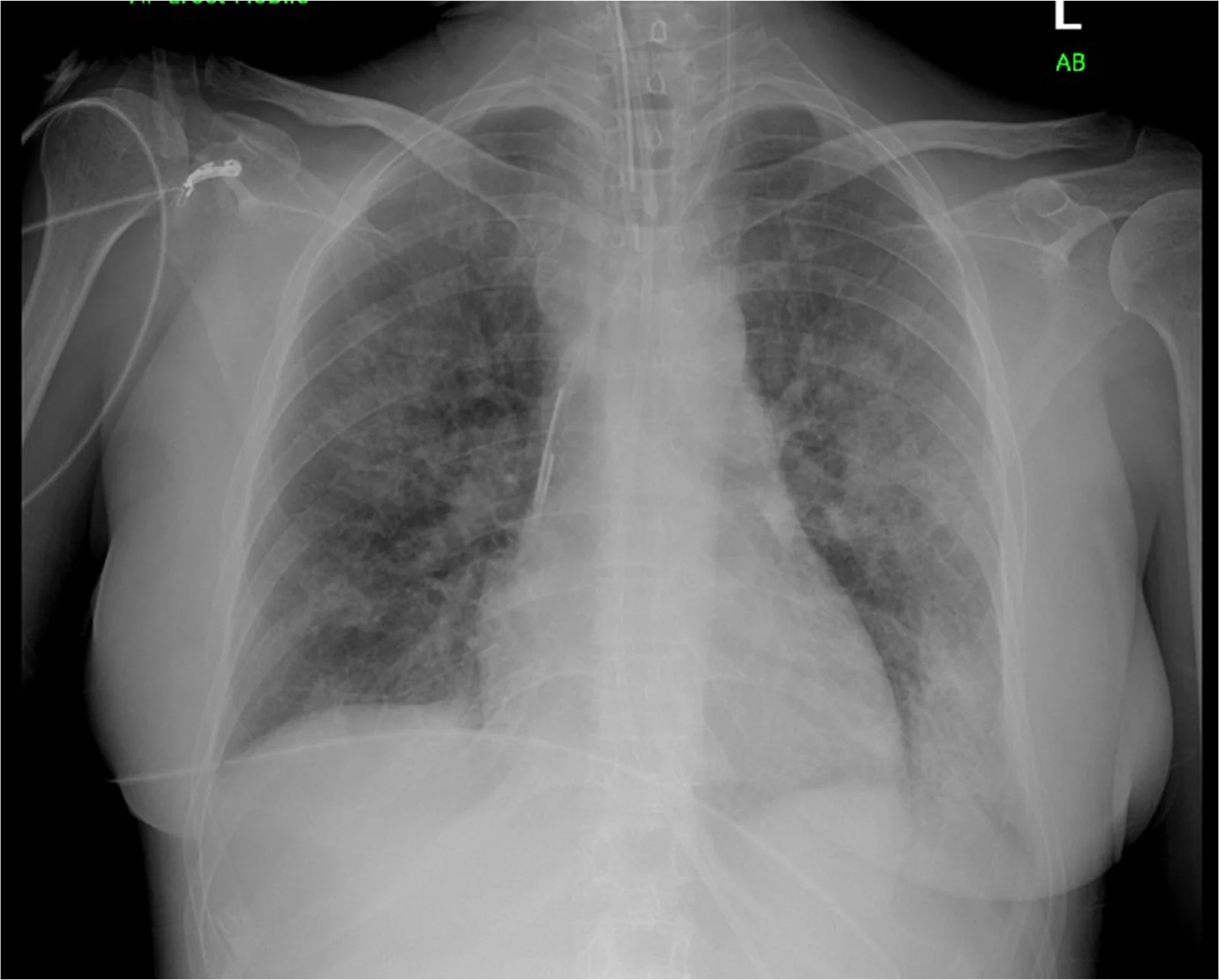
Chest X-ray revealing bilateral infiltrates suggestive of ARDS.

On day 3, H-score was 248 (Table 1) indicating > 99% probability of HLH [7], hence she received three pulses of 1 g methylprednisolone then was switched to Prednisolone 60 mg and Ciclosporin 5 mg/kg daily. Ferritin initially reduced to 8925ug/l (Fig. 1), however rebounded to 14235ug/L two days later with fluctuating consciousness, fever, hypoxia (saturations 88% on air) and CRP 235 mg/l. Repeat septic screen was negative but chest imaging revealed bilateral infiltrates suggestive of acute respiratory distress syndrome (Fig. 2). Anakinra 100 mg subcutaneous daily was added for HLH relapse and prednisolone switched to intravenous hydrocortisone 50 mg QDS in the context of fluctuating consciousness. Over the next 5 days, there was significant clinical and biochemical improvement.

**Table 1:** H-score for the diagnosis of HLH (adapted from Fardet et al) [5]. H-score > 241 (>99% probability), H-score < 90 (<1%), suspect HLH if H-score > 169. Our patient’s scores shown in bold: Total 248.

**Criterion**	**Result**	**Score**
Underlying immune suppression	No	0
	**Yes**	**18**
Temperature (°C)	<38.4	0
	**38.4–39.4**	**33**
	>39.4	49
Organomegaly	**None**	**0**
	Hepatomegaly or Splenomegaly	23
	Hepatomegaly and Splenomegaly	38
Cytopenias: Hb < 92 g/l WCC <5x10^9^/l Plt < 110 ×10^9^/l	1 line	0
	2 lines	24
	**3 lines**	**34**
Ferritin (ug/l)	<2000	0
	2000–6000	35
	**>6000**	**50**
Triglycerides (mmol/l)	<1.5	0
	1.5–4	44
	**>4**	**64**
Fibrinogen (g/l)	>2.5	0
	**<2.5**	**30**
AST (IU/l)	<30	0
	**>30**	**19**
Haemophagocytosis on bone marrow aspiration	**No**	**0**
	Yes	35

**Figure 3:**
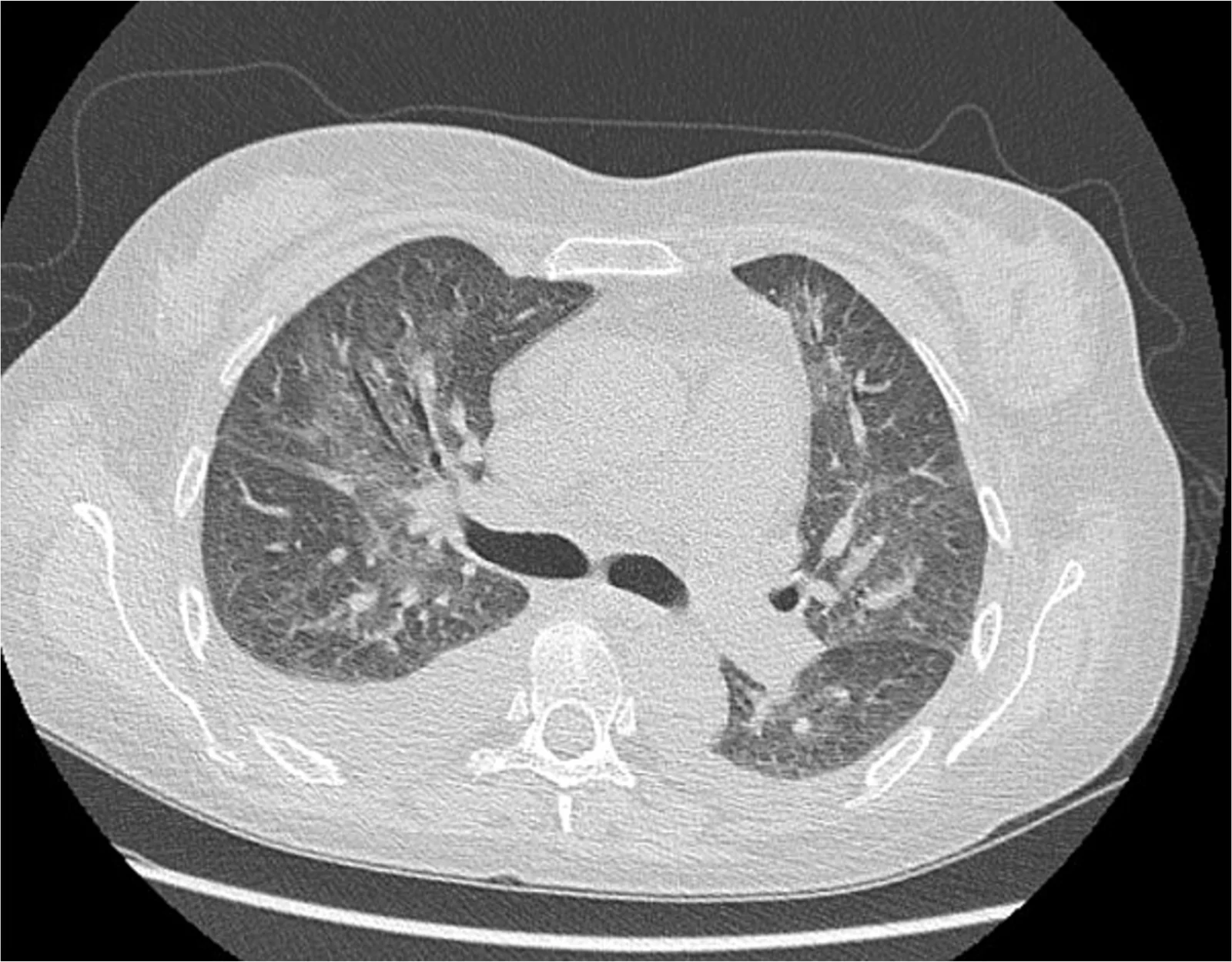
High resolution CT chest showing bilateral consolidation, atelectasis, ground-glass changes and bilateral pleural effusions.

However, after day 21, HLH markers worsened and she deteriorated with respiratory failure (Fig. 3) and Klebsiella septicaemia which was treated with intravenous gentamicin. IVIG 2 mg/kg was given for a second HLH relapse. Despite this, further rapid deterioration occurred with ferritin rising to 23599ug/l. Bone marrow aspiration confirmed haemophagocytosis without lymphoproliferation. After multidisciplinary discussion, Anakinra was increased to 500 mg daily (8 mg/kg). This produced a significant and sustained improvement, and she was stepped off intensive care on day 43.

Immunosuppression was gradually tapered, and she was eventually discharged on anakinra 400 mg daily, ciclosporin 2 mg/kg, mycophenolate 1 g twice-daily and prednisolone 10 mg daily. H-Score on discharge was 96 and ferritin 1591ug/l.

18 months from initial presentation, she remained well with no further relapses, ferritin 1027ug/L and SLEDAI score 0. She had weaned treatment to anakinra 100 mg every third day, prednisolone 2.5 mg daily and mycophenolate 1 g twice-daily, and was adherent with no adverse effects. Her mobility had normalised, and she felt able to perform all her daily activities.

This is the first reported case of an adult with SLE-induced HLH and additional viral trigger, achieving rapid, sustained remission with high-dose anakinra after only partially and transiently responding to conventional doses and other first-line immunosuppression.

## Discussion

Early recognition and treatment of HLH is vital to prevent multi-organ failure and death. Anakinra is a recombinant human interleukin-1 receptor antagonist; interleukin-1 is crucial in initiating and propagating hyperinflammation, hence its blockade is central in treating HLH. Despite the lack of controlled studies, it is widely used off-label as first-line alongside steroids [1]; efficacy is supported by case series with different underlying triggers, including SLE [8, 9]. Starting doses of 1–2 mg/kg daily have conventionally been used, increasing to 8 mg/kg if required [10], although earlier dose escalation is increasingly considered. In the cases described, higher doses were used later in the disease course, after failure of other treatments. Patients with active disease despite standard doses responded better to higher doses, achieving remission [8]. In some cases, remission occurred rapidly after weeks of incomplete response to first-line treatment including standard anakinra doses [9].

Our case details the effective use of anakinra in SLE-associated HLH, specifically the rapid and persistent efficacy of higher doses in the refractory stage, which facilitated sustained remission. This is consistent with other cases in which high-dose anakinra was effective following incomplete responses to lower doses, with ferritin as a crucial marker of response.

Given that choice of initial therapy should account for disease severity [3], we argue that high-dose anakinra should be considered immediately after steroid failure in cases that are particularly severe at presentation. Starting with higher doses then tapering later may be more effective than the stepwise escalation previously practiced. This would maximise the narrow window of opportunity to successfully treat these patients withvery high mortality.

Treatment was safely instituted in a DGH setting, even in the context of systemic infection. Its strong safety profile, and short half-life, make anakinra desirable in critically unwell patients with aggressive disease complicated by infection [3, 10].

Our case underlines the efficacy and safety of high-dose anakinra in severe treatment-refractory SLE-induced HLH, and supports its earlier use in such cases. This adds support to the shifting treatment paradigm in this direction [1, 3].

This is a single case, with Covid as a potential confounding trigger, and it should be noted that there is significant heterogeneity in severity and underlying triggers among described cases. Therefore we should exercise caution when drawing general conclusions about the wider population of HLH patients, and further long-term data is required.
